# Elimination of infectious HIV DNA by CRISPR–Cas9

**DOI:** 10.1016/j.coviro.2019.07.001

**Published:** 2019-08-23

**Authors:** Atze T Das, Caroline S Binda, Ben Berkhout

**Affiliations:** Laboratory of Experimental Virology, Department of Medical Microbiology, Amsterdam University Medical Centers, 1105AZ Amsterdam, The Netherlands

## Abstract

Current antiretroviral drugs can efficiently block HIV replication and prevent transmission, but do not target the HIV provirus residing in cells that constitute the viral reservoir. Because drug therapy interruption will cause viral rebound from this reservoir, HIV-infected individuals face lifelong treatment. Therefore, novel therapeutic strategies are being investigated that aim to permanently inactivate the proviral DNA, which may lead to a cure. Multiple studies showed that CRISPR–Cas9 genome editing can be used to attack HIV DNA. Here, we will focus on not only how this endonuclease attack can trigger HIV provirus inactivation, but also how virus escape occurs and this can be prevented.

## Genome editing strategies against HIV

**Figure 1:**
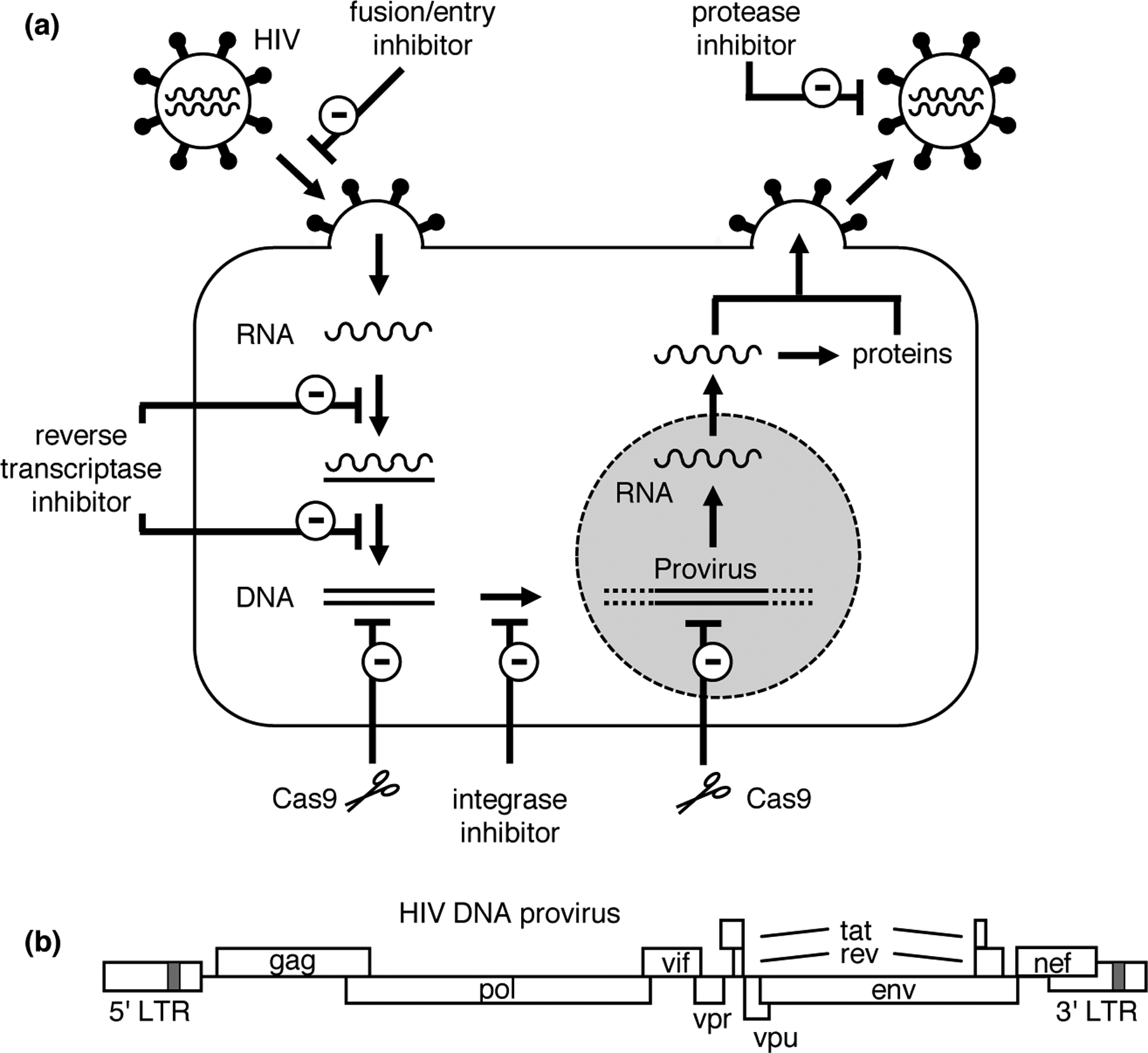
HIV-1 replication cycle and antiviral therapy. **(a)** The HIV particle contains two genomic RNA copies. The Env protein exposed at the viral membrane mediates attachment to the CD4 receptor and CCR5 or CXCR4 co-receptor of target T cells. Upon membrane fusion and virus entry, the viral RNA genome is reverse transcribed into DNA with a complete LTR at both ends. Upon integration into the cellular genome, this proviral DNA can be transcribed by the cellular RNA polymerase II transcription complex. RNA transcripts are processed by the cellular capping, polyadenylation and splicing machinery and subsequently translated. Genomic RNA dimers are packaged into new virus particles that assemble and bud at the cellular membrane. Antiviral drugs are grouped in six classes. Fusion inhibitors bind Env during the membrane fusion process, thus inhibiting virus entry. Entry inhibitors (CCR5 antagonists) bind CCR5 and inhibit entry of virus isolates that use the CCR5 co-receptor. Nucleoside reverse transcriptase inhibitors (NRTIs) and non-nucleoside reverse transcriptase inhibitors (NNRTIs) inhibit the viral RT enzyme. Integrase strand transfer inhibitors (INSTIs) target the viral integrase enzyme that is essential for the integration of the proviral DNA copy into the cellular genome. Protease inhibitors (PIs) inhibit the viral protease enzyme required for the processing of the Gag and Pol precursor proteins into the mature structural and enzymatic proteins. CRISPR–Cas9 nuclease can target and cleave the dsDNA that is formed upon reverse transcription of the viral RNA, either before or after integration into the cellular DNA. **(b)** The HIV-1 proviral DNA with nine open reading frames and the 5′ and 3′ LTRs. HIV transcription is driven by the 5′ LTR promoter. The approximately 9-kb long primary transcript is polyadenylated in the 3′ LTR region. The unspliced RNA is used as mRNA for the production of the structural (Gag) and enzymatic (Pol) proteins, and as genomic RNA (gRNA) that is packaged into virus particles. Differential splicing of the RNA transcripts yields mRNAs encoding the regulatory (Tat and Rev), accessory (Nef, Vif, Vpr) and envelope (Env) proteins. Reproduced (with minor modifications) from Wang et al. [24] (https://doi.org/10.1016/j.virusres.2017.07.020). © Wang *et al*. Distributed under the terms of the Creative Commons Attribution 4.0 International License (http://creativecommons.org/licenses/by/4.0/).

Although much progress has been made in the fight against HIV, the causative agent of acquired immune deficiency syndrome (AIDS), a definitive cure is still lacking. Multiple potent antiviral drugs have been developed that target different steps in the viral replication cycle (Figure 1a). Combining several drugs in a combination antiretroviral therapy (cART) can reduce the viral load in patients to undetectable levels and prevent disease progression. However, these drugs do not target the HIV proviral DNA present in viral reservoir cells. The latently infected reservoir cells, predominantly long-living resting T cells but also other cell types [1,2], are not detected by the immune system and the virus will rebound from this reservoir when therapy is interrupted. HIV-infected individuals therefore face lifelong cART, which forms a serious burden because of the strict adherence that is required to prevent development of drug resistance and the potential side effects of the drugs. Ideally, novel antiviral strategies would permanently inactivate or even remove the integrated proviral DNA in all infected cells. Several DNA editing tools have been developed in the past decade that open the way to such curative anti-HIV strategies.

**Figure 2:**
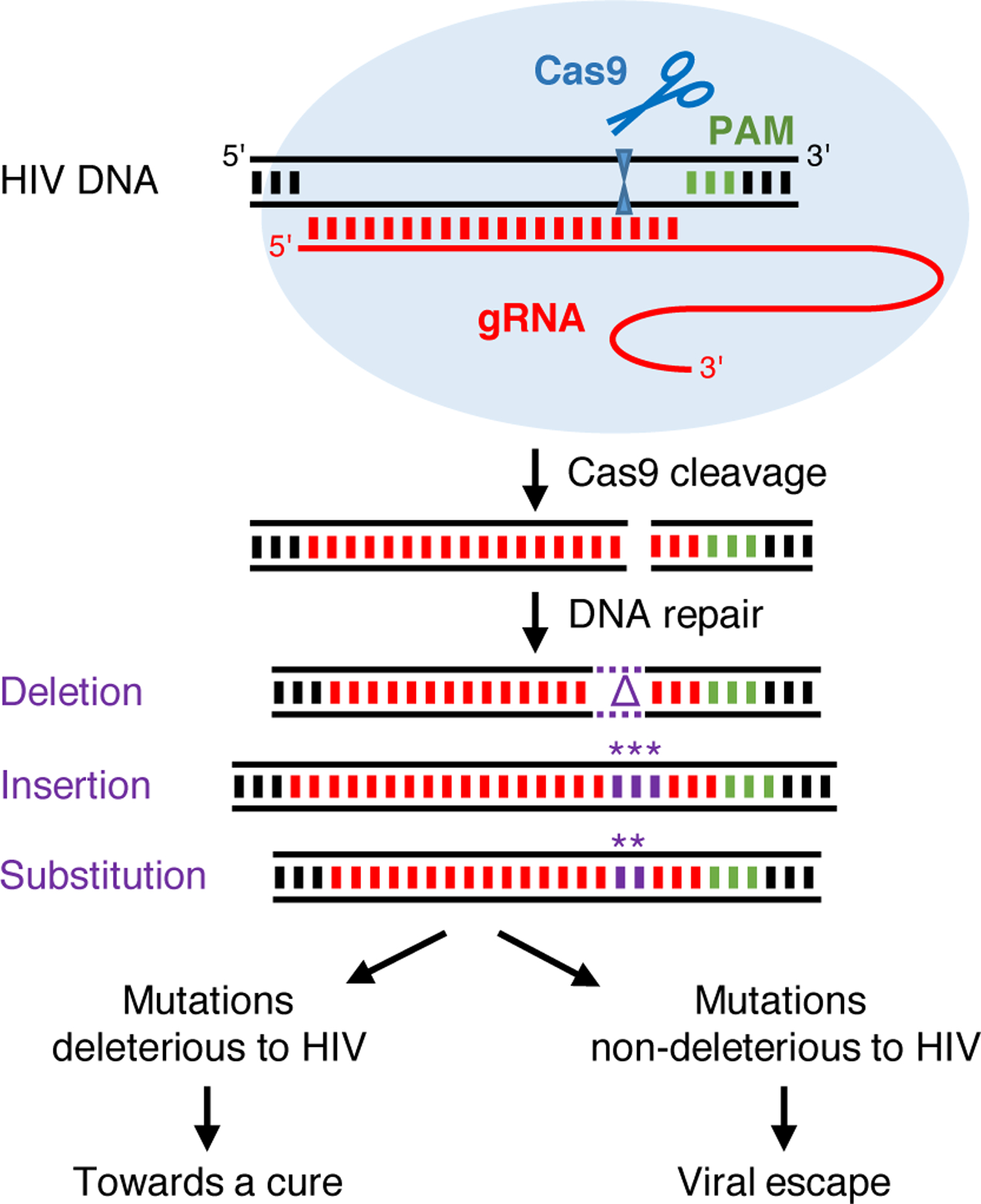
Cas9/gRNA attack of HIV DNA. Cas9 is directed to the HIV DNA by the gRNA and cleaves the target DNA at a position 3 nt from the PAM. The dsDNA break is repaired by the cellular DNA repair machinery (NHEJ and MMEJ) which results in nucleotide insertions, deletions and substitutions at the cleavage site. Most of these mutations will be deleterious and inactivate the virus, but some may be compatible with virus replication yet prevent gRNA recognition of the target site and thus lead to viral escape (PAM, protospacer adjacent motif; gRNA, guide RNA; * substituted or inserted nucleotides; Δ, deleted nucleotides). Reproduced (with minor modifications) from Liang et al. [42] (https://doi.org/10.1186/s12977-016-0270-0). © Liang *et al*. Distributed under the terms of the Creative Commons Attribution 4.0 International License (http://creativecommons.org/licenses/by/4.0/).

Initially, tailored Cre recombinases were proposed to excise the integrated proviral DNA from the cellular genome through recombination at the HIV long terminal repeat (LTR)that is present at both the 5′ and 3′ proviral DNA end (Figure 1b) [3–5]. The proviral DNA can also be targeted and disrupted by designer endonucleases that cleave-specific sequences in the viral DNA, such as zinc finger nucleases (ZFN), transcription activator-like effector nucleases (TALEN) and homing endonucleases [6–10]. More recently, CRISPR–Cas9 has become a very popular endonuclease to attack HIV DNA. This tool is derived from the CRISPR–Cas system that detects and cleaves nucleic acids from invading viruses and plasmids in bacteria and archaea [11–13]. The CRISPR-associated endonuclease Cas9 of *Streptococcus pyogenes* (spCas9) was developed into a genome editing tool that cleaves double-stranded (ds) DNA in eukaryotic cells. Sequence specificity is mediated by a 20 nucleotide (nt) sequence in the guide RNA (gRNA) that directs Cas9 to a complementary DNA target (Figure 2). Only complementary sequences flanked by a protospacer adjacent motif (PAM; NGG for spCas9) can be cleaved [14–17]. The dsDNA breaks resulting from Cas9 cleavage are repaired by cellular DNA repair mechanisms. These mechanisms include classical non-homologous end-joining (NHEJ), which ligates the DNA ends with frequent introduction of insertions or deletions (indels), and microhomology-mediated end-joining (MMEJ), in which short matching sequences present at the DNA ends anneal, eventually resulting in deletion of intervening nucleotides [18,19].

Simultaneous targeting of two distant loci can result in mutations at both gRNA target sites, but also in excision or inversion of the intervening sequences [20–22,23^•^]. For example, Canver *et al*. [23^•^] observed excisions and inversions in approximately 27% and 13% of sequences, respectively, when analyzing the Cas9-induced mutations resulting from several dual-gRNA combinations with an intervening region ranging from 2 to 20 kilobases (kb). Such excisions and inversions are frequently accompanied by indels at the cleavage sites. The CRISPR–Cas toolbox expanded in recent years and now includes systems originating from diverse bacterial species with distinct gRNA and PAM characteristics and different target specificities. The high specificity and efficiency of the CRISPR–Cas9 system have led to its widespread application, including in anti-HIV strategies [24]

## CRISPR–Cas9-targeting of HIV infection

The CRISPR–Cas9 system can be used to modify host cells in such a way that they are no longer susceptible to HIV infection. Inspired by the successful cure of the ‘Berlin patient’, who underwent allogeneic stem cell transplantation with donor cells lacking the CCR5 co-receptor, several studies focused on targeting the CCR5 gene [25^•^]. However, CCR5 inactivation may trigger envelope mutations that shift viral receptor usage from CCR5 to CXCR4 [26,27]. CRISPR–Cas9 can also target CXCR4 [28] or other cellular factors involved in HIV replication [29,30], but this may have undesirable side effects on cell physiology.

Instead of targeting host co-factors, the viral DNA can be targeted directly by introduction of the Cas9 protein and anti-viral gRNA in HIV-infected cells (Figure 1a).

Alternatively, cells can be harnessed with the CRISPR reagents to immediately attack and cleave the reverse transcribed viral DNA that is produced upon infection. Actually, the reported inhibition of virus replication in Cas9/gRNA harnessed cells is likely caused by the combined effects on HIV DNA before and after integration.

## Inhibition of virus replication and viral escape

Initial CRISPR–Cas9 studies involving replication-competent HIV demonstrated efficient inhibition of virus replication in short-term cell culture experiments. However, these studies did not address virus escape, even though HIV-1 is well-known for its capacity to develop resistance against inhibitors [31–33]. We and others therefore tested HIV replication in long-term cultures of T cells stably expressing Cas9 and an antiviral gRNA [34^••^,35^••^,^36^,^37^–^40^]. These studies confirmed that CRISPR–Cas9 can potently inhibit HIV replication, but also showed that the virus frequently escapes from this inhibition, which was due to acquired mutations clustering around the Cas9 cleavage site [34^••^]. Intriguingly, indels of variable size were observed in poorly conserved targets (e.g. in the LTR promoter region), whereas mostly substitutions and 3-nt insertions were found in conserved targets (e.g. in protein-coding domains). This mutational pattern differs strikingly from that observed upon virus escape from other inhibitors like ART drugs or RNA-interference therapeutics, where predominantly nucleotide substitutions are observed that are generated during the error-prone reverse transcription process [41]. The frequent indels that cluster at the Cas9 cleavage site implicate the cellular DNA repair mechanism that acts on the Cas9-generated dsDNA breaks in the generation of HIV escape viruses [42]. Upon Cas9 cleavage, the proviral DNA is repaired by cellular DNA repair pathways that introduce mutations (mostly indels, but also substitutions) at the cleavage site upstream of the PAM (Figure 2). Because this target domain is very important for gRNA/Cas9 binding, most mutations will prevent further Cas9 cleavage. In addition, they can also inactivate the virus (e.g. due to a frameshift mutation or inactivation of an essential RNA or protein domain), but some mutations will be compatible with virus replication, yet prevent gRNA binding, thus resulting in escape viruses. Wang *et al*. [35^••^] demonstrated that the escape mutations indeed originate from the cleaved and repaired proviral DNA pool, indicating that cellular DNA repair facilitated viral escape. However, a minor contribution of regular RT-generated mutations in virus escape cannot be excluded as some nucleotide substitutions were detected further away from the Cas9 cleavage site [34^••^,^36^]. Moreover, a poorly replicating escape virus resulting from Cas9 cleavage and subsequent DNA repair may accumulate additional RT-produced mutations in the target site or compensatory mutations elsewhere to increase its replication capacity.

In our study, the period of virus suppression varied for different gRNAs but did not correlate with the capacity of the gRNAs to induce HIV DNA cleavage and to suppress gene expression [34^••^]. Instead, the time to escape strongly correlated with the evolutionary conservation of the target sequence. Rapid viral escape was observed when poorly conserved HIV sequences were targeted, while escape was delayed when strongly conserved viral sequences were targeted. Poorly conserved HIV sequences correspond to non-essential regions, which can relatively easily accommodate the indels that are introduced during DNA repair. In contrast, highly conserved sequences correspond to essential viral regions, which will tolerate only specific mutations that are generated less frequently during DNA repair (e.g. nt substitutions and nt-triplet insertions that do not destroy the open reading frame).

## Combinatorial CRISPR–Cas9 attack prevents viral escape and triggers inactivation of the viral genome

These studies demonstrate that single gRNA/Cas9 targeting of HIV-1 can potently inhibit virus replication, but subsequent DNA repair facilitates virus escape. As previously shown when treating patients with antiviral drugs and when testing RNAi antivirals in cell culture experiments [43], combining antivirals does not only increase the magnitude of virus inhibition (because of additive or possibly even synergistic antiviral effects), but also the genetic threshold for development of resistance, as multiple mutations at different positions in the viral genome will be required.

To test whether gRNA combinations can similarly prevent viral escape, we and others evaluated HIV replication in T cells harnessed with CRISPR–Cas9 and different combinations of two gRNAs [38,44^••^]. Indeed, combinations inhibited viral replication more effectively than the corresponding single gRNAs, but viral escape was eventually apparent for most combinations due to acquisition of mutations with the typical Cas9/DNA repair signature in both targets. However, some gRNA combinations targeting highly conserved HIV sequences were found to completely block virus replication for the duration of our experiment, which lasted over four months [44^••^]. In these cultures, we observed the gradual disappearance of wild-type and point-mutated HIV sequences and gradual accumulation of indels and multiple-nucleotide substitutions at both target sites, indicating repeated CRISPR–Cas9 attack on point-mutated targets. Attempts to rescue replication-competent virus from the infected dual-gRNA protected cells by co-culturing with susceptible cells failed after some incubation period. These results demonstrated that the infected cells were functionally cured through mutation of both antiviral target sites, leaving the cells with a graveyard of inactivated HIV proviruses. These studies provide the proof of principle that CRISPR–Cas9 can be used to cure HIV-infected cells [44^••^].

## Mutation versus excision

**Figure 3:**
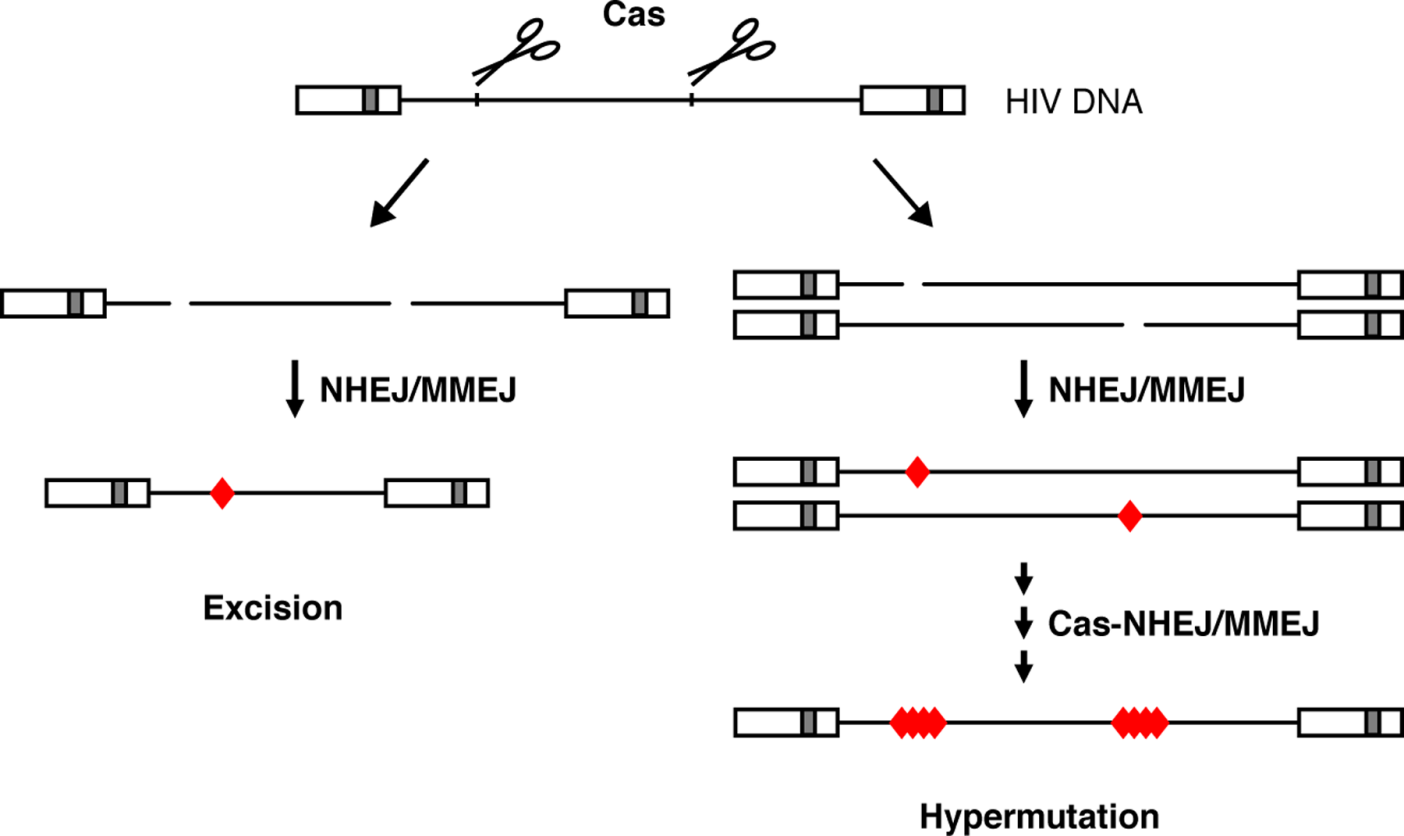
Excision or mutation of the HIV DNA. CRISPR–Cas9 attack of the HIV DNA with two gRNAs that target different viral domains (or with a single gRNA that targets both the 5′ and 3′ LTR domain) can result in excision (left panel) or dual-site mutation (right panel) of the viral DNA. Simultaneous cleavage at both targets and subsequent ligation of the free DNA ends will result in excision of the intervening fragment. Otherwise, for example when a DNA break is repaired before the second target is cleaved, both targets will be mutated. Wang *et al*. [44^••^] identified gRNA combinations targeting highly conserved essential sequences that durably blocked virus replication in infected T cell cultures. These gRNA combinations resulted in hypermutation of the viral DNA, that is, major indels and multiple-nucleotide substitutions at both targets increased over time at the expense of wild-type and point-mutated HIV sequences, which is likely due to repeated CRISPR–Cas9 attack on point-mutated targets (red diamond: mutation due to error-prone DNA repair).

It has previously been suggested to excise integrated HIV proviruses with CRISPR–Cas9 and two gRNAs or a single gRNA targeting both LTRs (Figure 3) [45–48,49^•^,^50^–^53^]. Some studies focused exclusively on this goal, apparently assuming that provirus excision is the major mechanism behind HIV inactivation [49^•^,^53^]. Excision will require simultaneous cleavage of both targets, followed by ‘ligation’ of the ends. As the cleavage kinetics may differ for different Cas9 targets, this timing requirement may be more easily fulfilled with a single gRNA targeting the identical sequence in the 5′ and 3′ LTR, although the chromosomal environment will differ and possibly influence the cleavage and repair processes. Several studies suggested efficient excision of the proviral genome [48,49^•^,^53^]. However, the PCR-based strategy used to detect excision strongly favors detection of the short excision product over the longer non-excised product. In fact, some studies did also detect a non-excised product, which may represent inactivated genomes with mutated target sites, but could also correspond to wild-type genomes. Unfortunately, the experimental systems used in these studies did not support massive HIV replication and did not allow testing for complete and permanent virus inactivation or virus escape. Such necessary assay conditions were met in our study and although we could also detect a low level of provirus excision, we demonstrated that complete virus inactivation coincided with mutation at both target sites (Figure 3). Thus, hypermutation seems a major mechanism for HIV inactivation, which is in agreement with the high frequency of dual-site mutations observed upon dual-gRNA cleavage in genome editing studies [20–22,23^•^]. Fragment inversion, detected at a low frequency in these genome editing studies, may also contribute to HIV inactivation.

## Countering HIV sequence variation

HIV demonstrates considerable genetic variation, with four phylogenetic groups (M, N, O, and P), multiple subtypes and much inter and intra-patient sequence diversity. Sequence variation in the gRNA target site may affect the Cas9 cleavage efficiency and thereby compromise the antiviral strategy. Single nucleotide mismatches between the gRNA and DNA target, in particular mismatches in the PAM-proximal region and non-consensus PAM nucleotides, did indeed reduce Cas9 cleavage activity in studies that yielded algorithms to predict the activity of mismatching gRNAs [17,54].

Dampier *et al*. used such an algorithm to calculate the activity of published gRNAs against diverse HIV isolates [55] and to design personalized and broad-spectrum gRNA combinations based on within-patient sequence variants and consensus sequences from multiple patients, respectively [56]. This *in silico* analysis, for example, suggested that the gRNAs in our sterilizing dual-gRNA combinations were effective against 82–95% of all HIV-1 subtype B variants [56]. However, this estimation uses an arbitrary level of cleavage activity required for virus inactivation and the algorithm is based on experimental data from single-nt mismatches only and assumes that dual-nt mutations have a multiplicative effect. Roychoudhury *et al*. demonstrated that there was only a ‘trend to weak positive correlation’ between the *in silico* predicted and experimentally measured activity of gRNAs, when testing the knockdown activity of 59 LTR-targeting gRNAs in an LTR-GFP reporter assay [57]. By modeling the reservoir depletion during CRISPR–Cas9 therapy, these authors illustrate that reduced gRNA activity and limited coverage of the patient’s viral quasispecies will reduce the efficacy of the CRISPR–Cas9 therapy. However, our long-term virus escape experiments demonstrated that durable virus inhibition does not correlate with gRNA/Cas9 cleavage activity but rather with sequence conservation of the target sequence, which correlates inversely with the mutational escape options for the virus [34^••^]. In the CRISPR–Cas9 therapy, escape variants can be instantly produced due to Cas9 cleavage and subsequent DNA repair at the gRNA target site. Although continuation of ART treatment during CRISPR–Cas9 therapy will block virus replication and prevent reverse transcription-driven evolution, it will not prevent the generation of Cas9-induced mutations and thereby the possible formation of gRNA/Cas9-resistant variants. Such escape variants may lead to virus rebound upon discontinuation of ART. It is thus critically important that the gRNAs used in the CRISPR–Cas9 therapy do not only inactivate most, preferably all, replication-competent proviral genomes in the latent reservoir, but also that the genetic threshold to escape is high. Combination of gRNAs that simultaneously target multiple highly conserved sequences seems the best strategy.

We recently evaluated the impact of HIV genetic diversity on CRISPR–Cas9 antiviral activity and viral escape by testing the most effective dual-gRNA combinations against distinct HIV-1 isolates, including different subtypes [58^•^]. Despite the fact that the gRNAs were designed to target highly conserved viral sequences, these sites could mismatch at 1 or 2 nt-positions. Replication of nearly all isolates could be prevented by at least one gRNA combination, which caused inactivation of the proviral genomes and the gradual loss of replication-competent virus over time. Inspection of the gRNA targets in viruses that can be blocked versus those that cannot did shed light on the sequence requirements for an effective gRNA attack. Most 1-nt mismatches did not significantly affect gRNA/Cas9 inhibition, but the gRNA lost activity when the mismatch was positioned at the Cas9 cleavage site. In contrast, two mismatches — independent of the position in the target — significantly reduced the antiviral effect. Inclusion of such a non-effective gRNA turned the dual-gRNA therapy essentially into a single gRNA therapy from which the virus was able to escape. This study demonstrates that even minor sequence variation in conserved viral targets can affect the efficacy of the combinatorial CRISPR–Cas9 therapy. Unfortunately, the *in silico* predicted cleavage activity of the mismatching gRNAs as based on the above described algorithms [17,54] did not correlate with their capacity to durably inhibit virus replication and are thus poor predictors. Successful HIV cure attempts may therefore require elaborate testing of gRNAs.

## Future directions

CRISPR–Cas9 attack of the HIV proviral DNA in infected cells can lead to permanent inactivation of the virus when gRNA combinations are used that target essential, highly conserved viral domains. Besides coping with HIV genetic diversity, several other issues need to be addressed for the development of a safe and effective CRISPR–Cas9 HIV therapy. First, off-target CRISPR–Cas9 effects need to be excluded. Although *in silico* design tools can predict off-target sites and optimized CRISPR–Cas9 systems with increased sequence-specificity have been developed [59,60], experimental validation seems necessary to exclude mutation of non-target sequences in the human genome. Large deletions extending over many kb and complex genomic rearrangements have also been detected in Cas9 studies [22,61]. The frequency and potential harmful effects of such dramatic genome rearrangements (e.g. oncogene induction or tumor-suppressor gene disruption) need further investigation.

A sterilizing cure will require delivery of Cas9 and the gRNAs to all HIV-1 reservoir cells. Several methods are available for transient delivery of these components (e.g. gRNA–Cas9 ribonucleoprotein particles and virus-like particles [62–64]), but their *in vivo* delivery efficiency is likely suboptimal. Vectors based on adeno-associated virus (AAV) and HIV (lentiviral vector, LV) will facilitate prolonged Cas9 and gRNA activity and a more sustained therapeutic effect, but may also increase the risk of off-target effects. Although animal experiments show that HIV sequences can be targeted *in vivo* through AAV and LV-mediated delivery of the CRISPR reagents [49^•^,^53^,65^•^], the efficiency of current viral vectors is likely too low to reach all reservoir cells. A significant constraint is the restricted packaging capacity of the AAV and LV vectors, especially given the large size of the Cas9 gene. This problem may be reduced by the use of smaller Cas9 variants (e.g. *Staphylococcus aureus* Cas9 [saCas9]), truncated Cas9 proteins lacking non-essential domains or smaller gRNA/Cas9 cassettes [50,53,66,67]. The Cpf1 (Cas12a) system forms an interesting alternative as it has increased specificity but a small size, which could alleviate both the delivery and off-target problems [68,69]. The viral vector should preferably only target HIV reservoir cells, but development of such a vector is complicated by the fact that the viral reservoir is still poorly defined. Immune responses against the non-human Cas9 protein and the viral particles may also complicate this *in vivo* inactivation strategy [70,71].

CRISPR–Cas9 can be combined with other anti-HIV therapeutics such as antiviral drugs or RNAi molecules. The combinatorial approach will further reduce the level of virus replication, but also increase the genetic threshold for virus escape to occur. A combined CRISPR–Cas9 and RNAi attack on HIV, targeting both the viral DNA and RNA, did indeed inhibit HIV replication more durably than the corresponding monotherapies [72]. Furthermore, Dash *et al*. recently demonstrated that sequential treatment of HIV-infected humanized mice with ‘long-acting slow-effective release’ (LASER) ART using fatty-acid modified drugs and CRISPR–Cas9 using an AAV dual-gRNA saCas9 vector resulted in viral clearance and prevented viral rebound in ~40% of the treated animals, whereas viral rebound was observed in all animals that received monotherapy [73^•^].

## References

[1] Siliciano JD, Kajdas J, Finzi D, Quinn TC, Chadwick K, Margolick JB, Kovacs C, Gange SJ, Siliciano RF: Long-term follow-up studies confirm the stability of the latent reservoir for HIV-1 in resting CD4+ T cells. Nat Med 2003, 9:727–728. 12754504 10.1038/nm880

[2] Blankson JN, Persaud D, Siliciano RF: The challenge of viral reservoirs in HIV-1 infection. Annu Rev Med 2002, 53:557–593. 11818490 10.1146/annurev.med.53.082901.104024

[3] Sarkar I, Hauber I, Hauber J, Buchholz F: HIV-1 proviral DNA excision using an evolved recombinase. Science 2007, 316:1912–1915. 17600219 10.1126/science.1141453

[4] Karpinski J, Hauber I, Chemnitz J, Schafer C, Paszkowski-Rogacz M, Chakraborty D, Beschorner N, Hofmann-Sieber H, Lange UC, Grundhoff A : Directed evolution of a recombinase that excises the provirus of most HIV-1 primary isolates with high specificity. Nat Biotechnol 2016, 34:401–409. 26900663 10.1038/nbt.3467

[5] Buchholz F, Hauber J: In vitro evolution and analysis of HIV-1 LTR-specific recombinases. Methods 2011, 53:102–109. 20600935 10.1016/j.ymeth.2010.06.014

[6] Manjunath N, Yi G, Dang Y, Shankar P: Newer gene editing technologies toward HIV gene therapy. Viruses 2013, 5:2748–2766. 24284874 10.3390/v5112748 PMC3856413

[7] Benjamin R, Berges BK, Solis-Leal A, Igbinedion O, Strong CL, Schiller MR: TALEN gene editing takes aim on HIV. Hum Genet 2016, 135:1059–1070. 27170155 10.1007/s00439-016-1678-2 PMC5002248

[8] Stone D, Kiem HP, Jerome KR: Targeted gene disruption to cure HIV. Curr Opin HIV AIDS 2013, 8:217–223. 23478911 10.1097/COH.0b013e32835f736c PMC4226633

[9] Jerome KR: Disruption or excision of provirus as an approach to HIV cure. AIDS Patient Care STDS 2016, 30:551–555. 27855263 10.1089/apc.2016.0232 PMC5144885

[10] Stone D, Niyonzima N, Jerome KR: Genome editing and the next generation of antiviral therapy. Hum Genet 2016, 135:1071–1082. 27272125 10.1007/s00439-016-1686-2 PMC5002242

[11] Makarova KS, Haft DH, Barrangou R, Brouns SJ, Charpentier E, Horvath P, Moineau S, Mojica FJ, Wolf YI, Yakunin AF : Evolution and classification of the CRISPR-Cas systems. Nat Rev Microbiol 2011, 9:467–477. 21552286 10.1038/nrmicro2577 PMC3380444

[12] Gasiunas G, Barrangou R, Horvath P, Siksnys V: Cas9-crRNA ribonucleoprotein complex mediates specific DNA cleavage for adaptive immunity in bacteria. Proc Natl Acad Sci U S A 2012, 109:E2579–2586. 22949671 10.1073/pnas.1208507109 PMC3465414

[13] Wiedenheft B, Sternberg SH, Doudna JA: RNA-guided genetic silencing systems in bacteria and archaea. Nature 2012, 482:331–338. 22337052 10.1038/nature10886

[14] Cho SW, Kim S, Kim JM, Kim JS: Targeted genome engineering in human cells with the Cas9 RNA-guided endonuclease. Nat Biotechnol 2013, 31:230–232. 23360966 10.1038/nbt.2507

[15] Cong L, Ran FA, Cox D, Lin S, Barretto R, Habib N, Hsu PD, Wu X, Jiang W, Marraffini LA : Multiplex genome engineering using CRISPR/Cas systems. Science 2013, 339:819–823. 23287718 10.1126/science.1231143 PMC3795411

[16] Hsu PD, Lander ES, Zhang F: Development and applications of CRISPR–Cas9 for genome engineering. Cell 2014, 157:1262–1278. 24906146 10.1016/j.cell.2014.05.010 PMC4343198

[17] Hsu PD, Scott DA, Weinstein JA, Ran FA, Konermann S, Agarwala V, Li Y, Fine EJ, Wu X, Shalem O : DNA targeting specificity of RNA-guided Cas9 nucleases. Nat Biotechnol 2013, 31:827–832. 23873081 10.1038/nbt.2647 PMC3969858

[18] Shen MW, Arbab M, Hsu JY, Worstell D, Culbertson SJ, Krabbe O, Cassa CA, Liu DR, Gifford DK, Sherwood RI: Predictable and precise template-free CRISPR editing of pathogenic variants. Nature 2018, 563:646–651. 30405244 10.1038/s41586-018-0686-x PMC6517069

[19] Allen F, Crepaldi L, Alsinet C, Strong AJ, Kleshchevnikov V, De Angeli P, Palenikova P, Khodak A, Kiselev V, Kosicki M : Predicting the mutations generated by repair of Cas9-induced double-strand breaks. Nat Biotechnol 2019, 37:64–72. 10.1038/nbt.4317 PMC6949135 30480667

[20] Boroviak K, Doe B, Banerjee R, Yang F, Bradley A: Chromosome engineering in zygotes with CRISPR/Cas9. Genesis 2016, 54:78–85. 26742453 10.1002/dvg.22915 PMC4819711

[21] Zhang L, Jia R, Palange NJ, Satheka AC, Togo J, An Y, Humphrey M, Ban L, Ji Y, Jin H : Large genomic fragment deletions and insertions in mouse using CRISPR/Cas9. PLoS One 2015, 10:e0120396. 25803037 10.1371/journal.pone.0120396 PMC4372442

[22] Shin HY, Wang C, Lee HK, Yoo KH, Zeng X, Kuhns T, Yang CM, Mohr T, Liu C, Hennighausen L: CRISPR/Cas9 targeting events cause complex deletions and insertions at 17 sites in the mouse genome. Nat Commun 2017, 8:15464. 28561021 10.1038/ncomms15464 PMC5460021

[R23] Canver MC, Bauer DE, Dass A, Yien YY, Chung J, Masuda T, Maeda T, Paw BH, Orkin SH: Characterization of genomic deletion efficiency mediated by clustered regularly interspaced short palindromic repeats (CRISPR)/Cas9 nuclease system in mammalian cells. J Biol Chem 2017, 292:2556. 28188222 10.1074/jbc.A114.564625 PMC5313121

[24] Wang G, Zhao N, Berkhout B, Das AT: CRISPR-Cas based antiviral strategies against HIV-1. Virus Res 2018, 244:321–332. 28760348 10.1016/j.virusres.2017.07.020

[R25] Allers K, Schneider T: CCR5Delta32 mutation and HIV infection: basis for curative HIV therapy. Curr Opin Virol 2015, 14:24–29. 26143158 10.1016/j.coviro.2015.06.007

[26] Fatkenheuer G, Nelson M, Lazzarin A, Konourina I, Hoepelman AI, Lampiris H, Hirschel B, Tebas P, Raffi F, Trottier B : Subgroup analyses of maraviroc in previously treated R5 HIV-1 infection. N Engl J Med 2008, 359:1442–1455. 18832245 10.1056/NEJMoa0803154

[27] Verheyen J, Thielen A, Lubke N, Dirks M, Widera M, Dittmer U, Kordelas L, Daumer M, de Jong DCM, Wensing AMJ : Rapid rebound of a preexisting CXCR4-tropic human immunodeficiency virus variant after allogeneic transplantation with CCR5 Delta32 homozygous stem cells. Clin Infect Dis 2019, 68:684–687. 30020413 10.1093/cid/ciy565

[28] Hou P, Chen S, Wang S, Yu X, Chen Y, Jiang M, Zhuang K, Ho W, Hou W, Huang J : Genome editing of CXCR4 by CRISPR/ cas9 confers cells resistant to HIV-1 infection. Sci Rep 2015, 5:15577. 26481100 10.1038/srep15577 PMC4612538

[29] Hultquist JF, Schumann K, Woo JM, Manganaro L, McGregor MJ, Doudna J, Simon V, Krogan NJ, Marson A: A Cas9 ribonucleoprotein platform for functional genetic studies of HIV-host interactions in primary human T cells. Cell Rep 2016, 17:1438–1452. 27783955 10.1016/j.celrep.2016.09.080 PMC5123761

[30] Park RJ, Wang T, Koundakjian D, Hultquist JF, Lamothe-Molina P, Monel B, Schumann K, Yu H, Krupzcak KM, Garcia-Beltran W : A genome-wide CRISPR screen identifies a restricted set of HIV host dependency factors. Nat Genet 2017, 49:193–203. 27992415 10.1038/ng.3741 PMC5511375

[31] Boden D, Pusch O, Lee F, Tucker L, Ramratnam B: Human immunodeficiency virus type 1 escape from RNA interference. J Virol 2003, 77:11531–11535. 14557638 10.1128/JVI.77.21.11531-11535.2003 PMC229317

[32] Das AT, Brummelkamp TR, Westerhout EM, Vink M, Madiredjo M, Bernards R, Berkhout B: Human immunodeficiency virus type 1 escapes from RNA interference-mediated inhibition. J Virol 2004, 78:2601–2605. 14963165 10.1128/JVI.78.5.2601-2605.2004 PMC369246

[33] Westerhout EM, Ooms M, Vink M, Das AT, Berkhout B: HIV-1 can escape from RNA interference by evolving an alternative structure in its RNA genome. Nucleic Acids Res 2005, 33:796–804. 15687388 10.1093/nar/gki220 PMC548362

[R34] Wang G, Zhao N, Berkhout B, Das AT: CRISPR-Cas9 can inhibit HIV-1 replication but NHEJ repair facilitates virus escape. Mol Ther 2016, 24:522–526. 26796669 10.1038/mt.2016.24 PMC4786927

[R35] Wang Z, Pan Q, Gendron P, Zhu W, Guo F, Cen S, Wainberg MA, Liang C: CRISPR/Cas9-derived mutations both inhibit HIV-1 replication and accelerate viral escape. Cell Rep 2016, 15:481–489. 27068471 10.1016/j.celrep.2016.03.042

[36] Yoder KE, Bundschuh R: Host double strand break repair generates HIV-1 strains resistant to CRISPR/Cas9. Sci Rep 2016, 6:29530. 27404981 10.1038/srep29530 PMC4941621

[37] Ueda S, Ebina H, Kanemura Y, Misawa N, Koyanagi Y: Anti-HIV-1 potency of the CRISPR/Cas9 system insufficient to fully inhibit viral replication. Microbiol Immunol 2016, 60:483–496. 27278725 10.1111/1348-0421.12395

[38] Lebbink RJ, de Jong DC, Wolters F, Kruse EM, van Ham PM, Wiertz EJ, Nijhuis M: A combinational CRISPR/Cas9 gene-editing approach can halt HIV replication and prevent viral escape. Sci Rep 2017, 7:41968. 28176813 10.1038/srep41968 PMC5296774

[39] Mefferd AL, Bogerd HP, Irwan ID, Cullen BR: Insights into the mechanisms underlying the inactivation of HIV-1 proviruses by CRISPR/Cas. Virology 2018, 520:116–126. 29857168 10.1016/j.virol.2018.05.016 PMC6100742

[40] Wang Z, Wang W, Cui YC, Pan Q, Zhu W, Gendron P, Guo F, Cen S, Witcher M, Liang C: HIV-1 employs multiple mechanisms to resist Cas9/single guide RNA targeting the viral primer binding site. J Virol 2018, 92. 10.1128/JVI.01135-18 PMC6158435 30068653

[41] von Eije KJ, ter Brake O, Berkhout B: Human immunodeficiency virus type 1 escape is restricted when conserved genome sequences are targeted by RNA interference. J Virol 2008, 82:2895–2903. 18077712 10.1128/JVI.02035-07 PMC2258968

[42] Liang C, Wainberg MA, Das AT, Berkhout B: CRISPR/Cas9: a double-edged sword when used to combat HIV infection. Retrovirology 2016, 13:37. 27230886 10.1186/s12977-016-0270-0 PMC4882869

[43] ter Brake O, Konstantinova P, Ceylan M, Berkhout B: Silencing of HIV-1 with RNA interference: a multiple shRNA approach. Mol Ther 2006, 14:883–892. 16959541 10.1016/j.ymthe.2006.07.007

[R44] Wang G, Zhao N, Berkhout B, Das AT: A combinatorial CRISPR-Cas9 attack on HIV-1 DNA extinguishes all infectious provirus in infected T cell cultures. Cell Rep 2016, 17:2819–2826. 27974196 10.1016/j.celrep.2016.11.057

[45] Ebina H, Misawa N, Kanemura Y, Koyanagi Y: Harnessing the CRISPR/Cas9 system to disrupt latent HIV-1 provirus. Sci Rep 2013, 3:2510. 23974631 10.1038/srep02510 PMC3752613

[46] Hu W, Kaminski R, Yang F, Zhang Y, Cosentino L, Li F, Luo B, Alvarez-Carbonell D, Garcia-Mesa Y, Karn J : RNA-directed gene editing specifically eradicates latent and prevents new HIV-1 infection. Proc Natl Acad Sci U S A 2014, 111:11461–11466. 25049410 10.1073/pnas.1405186111 PMC4128125

[47] Liao HK, Gu Y, Diaz A, Marlett J, Takahashi Y, Li M, Suzuki K, Xu R, Hishida T, Chang CJ : Use of the CRISPR/Cas9 system as an intracellular defense against HIV-1 infection in human cells. Nat Commun 2015, 6:6413. 25752527 10.1038/ncomms7413

[48] Kaminski R, Chen Y, Fischer T, Tedaldi E, Napoli A, Zhang Y, Karn J, Hu W, Khalili K: Elimination of HIV-1 genomes from human T-lymphoid cells by CRISPR/Cas9 gene editing. Sci Rep 2016, 6:22555. 26939770 10.1038/srep22555 PMC4778041

[R49] Kaminski R, Bella R, Yin C, Otte J, Ferrante P, Gendelman HE, Li H, Booze R, Gordon J, Hu W : Excision of HIV-1 DNA by gene editing: a proof-of-concept in vivo study. Gene Ther 2016, 23:690–695. 27194423 10.1038/gt.2016.41 PMC4974122

[50] Ran FA, Cong L, Yan WX, Scott DA, Gootenberg JS, Kriz AJ, Zetsche B, Shalem O, Wu X, Makarova KS : In vivo genome editing using Staphylococcus aureus Cas9. Nature 2015, 520:186–191. 25830891 10.1038/nature14299 PMC4393360

[51] Yin C, Zhang T, Li F, Yang F, Putatunda R, Young WB, Khalili K, Hu W, Zhang Y: Functional screening of guide RNAs targeting the regulatory and structural HIV-1 viral genome for a cure of AIDS. AIDS 2016, 30:1163–1174. 26990633 10.1097/QAD.0000000000001079 PMC4851589

[52] Dampier W, Nonnemacher MR, Sullivan NT, Jacobson JM, Wigdahl B: HIV excision utilizing CRISPR/Cas9 technology: attacking the proviral quasispecies in reservoirs to achieve a cure. MOJ Immunol 2014, 1. 10.15406/moji.2014.01.00022 PMC4399856 25893217

[53] Yin C, Zhang T, Qu X, Zhang Y, Putatunda R, Xiao X, Li F, Xiao W, Zhao H, Dai S : In vivo excision of HIV-1 provirus by saCas9 and multiplex single-guide RNAs in animal models. Mol Ther 2017, 25:1168–1186. 28366764 10.1016/j.ymthe.2017.03.012 PMC5417847

[54] Doench JG, Fusi N, Sullender M, Hegde M, Vaimberg EW, Donovan KF, Smith I, Tothova Z, Wilen C, Orchard R : Optimized sgRNA design to maximize activity and minimize off-target effects of CRISPR-Cas9. Nat Biotechnol 2016, 34:184–191. 26780180 10.1038/nbt.3437 PMC4744125

[55] Dampier W, Sullivan NT, Chung CH, Mell JC, Nonnemacher MR, Wigdahl B: Designing broad-spectrum anti-HIV-1 gRNAs to target patient-derived variants. Sci Rep 2017, 7:14413. 29089503 10.1038/s41598-017-12612-z PMC5663707

[56] Dampier W, Sullivan NT, Mell JC, Pirrone V, Ehrlich GD, Chung CH, Allen AG, DeSimone M, Zhong W, Kercher K : Broad-spectrum and personalized guide RNAs for CRISPR/Cas9 HIV-1 therapeutics. AIDS Res Hum Retroviruses 2018, 34:950–960. 29968495 10.1089/aid.2017.0274 PMC6238604

[57] Roychoudhury P, De Silva Feelixge H, Reeves D, Mayer BT, Stone D, Schiffer JT, Jerome KR: Viral diversity is an obligate consideration in CRISPR/Cas9 designs for targeting the HIV reservoir. BMC Biol 2018, 16:75. 29996827 10.1186/s12915-018-0544-1 PMC6040082

[R58] Darcis G, Binda CS, Klaver B, Herrera-Carrillo E, Berkhout B, Das AT: The impact of HIV-1 genetic diversity on CRISPR-Cas9 antiviral activity and viral escape. Viruses 2019, 11. 10.3390/v11030255 PMC6466431 30871200

[59] Ryan DE, Taussig D, Steinfeld I, Phadnis SM, Lunstad BD, Singh M, Vuong X, Okochi KD, McCaffrey R, Olesiak M : Improving CRISPR-Cas specificity with chemical modifications in single-guide RNAs. Nucleic Acids Res 2018, 46:792–803. 29216382 10.1093/nar/gkx1199 PMC5778453

[60] Tycko J, Myer VE, Hsu PD: Methods for optimizing CRISPR-Cas9 genome editing specificity. Mol Cell 2016, 63:355–370. 27494557 10.1016/j.molcel.2016.07.004 PMC4976696

[61] Kosicki M, Tomberg K, Bradley A: Repair of double-strand breaks induced by CRISPR-Cas9 leads to large deletions and complex rearrangements. Nat Biotechnol 2018, 36:765–771. 30010673 10.1038/nbt.4192 PMC6390938

[62] Choi JG, Dang Y, Abraham S, Ma H, Zhang J, Guo H, Cai Y, Mikkelsen JG, Wu H, Shankar P : Lentivirus pre-packed with Cas9 protein for safer gene editing. Gene Ther 2016, 23:627–633. 27052803 10.1038/gt.2016.27

[63] Campbell LA, Coke LM, Richie CT, Fortuno LV, Park AY, Harvey BK: Gesicle-mediated delivery of CRISPR/Cas9 ribonucleoprotein complex for inactivating the HIV provirus. Mol Ther 2019, 27:151–163. 30389355 10.1016/j.ymthe.2018.10.002 PMC6318701

[64] Montagna C, Petris G, Casini A, Maule G, Franceschini GM, Zanella I, Conti L, Arnoldi F, Burrone OR, Zentilin L : VSV-G-enveloped vesicles for traceless delivery of CRISPR-Cas9. Mol Ther Nucleic Acids 2018, 12:453–462. 30195783 10.1016/j.omtn.2018.05.010 PMC6041463

[R65] Bella R, Kaminski R, Mancuso P, Young WB, Chen C, Sariyer R, Fischer T, Amini S, Ferrante P, Jacobson JM : Removal of HIV DNA by CRISPR from patient blood engrafts in humanized mice. Mol Ther Nucleic Acids 2018, 12:275–282. 30195766 10.1016/j.omtn.2018.05.021 PMC6011019

[66] Wang L, Li F, Dang L, Liang C, Wang C, He B, Liu J, Li D, Wu X, Xu X : In vivo delivery systems for therapeutic genome editing. Int J Mol Sci 2016, 17. 10.3390/ijms17050626 PMC4881452 27128905

[67] Gao Z, Herrera-Carrillo E, Berkhout B: A single H1 promoter can drive both guide RNA and endonuclease expression in the CRISPR-Cas9 system. Mol Ther Nucleic Acids 2018, 14:32–40. 30530211 10.1016/j.omtn.2018.10.016 PMC6288460

[68] Bayat H, Modarressi MH, Rahimpour A: The conspicuity of CRISPR-Cpf1 system as a significant breakthrough in genome editing. Curr Microbiol 2018, 75:107–115. 29189942 10.1007/s00284-017-1406-8

[69] Gao Z, Herrera-Carrillo E, Berkhout B: Improvement of the CRISPR-Cpf1 system with ribozyme-processed crRNA. RNA Biol 2018, 15:1458–1467. 30470168 10.1080/15476286.2018.1551703 PMC6333430

[70] Wang D, Mou H, Li S, Li Y, Hough S, Tran K, Li J, Yin H, Anderson DG, Sontheimer EJ : Adenovirus-mediated somatic genome editing of Pten by CRISPR/Cas9 in mouse liver in spite of Cas9-specific immune responses. Hum Gene Ther 2015, 26:432–442. 26086867 10.1089/hum.2015.087 PMC4509492

[71] Chew WL, Tabebordbar M, Cheng JK, Mali P, Wu EY, Ng AH, Zhu K, Wagers AJ, Church GM: A multifunctional AAV-CRISPR-Cas9 and its host response. Nat Methods 2016, 13:868–874. 27595405 10.1038/nmeth.3993 PMC5374744

[72] Zhao N, Wang G, Das AT, Berkhout B: Combinatorial CRISPR-Cas9 and RNA interference attack on HIV-1 DNA and RNA can lead to cross-resistance. Antimicrob Agents Chemother 2017, 61. 10.1128/AAC.01486-17 PMC5700367 28893790

[R73] Dash PK, Kaminski R, Bella R, Su H, Mathews S, Ahooyi TM, Chen C, Mancuso P, Sariyer R, Ferrante P : Sequential LASER ART and CRISPR treatments eliminate HIV-1 in a subset of infected humanized mice. Nat Commun 2019, 10:2753. 31266936 10.1038/s41467-019-10366-y PMC6606613

